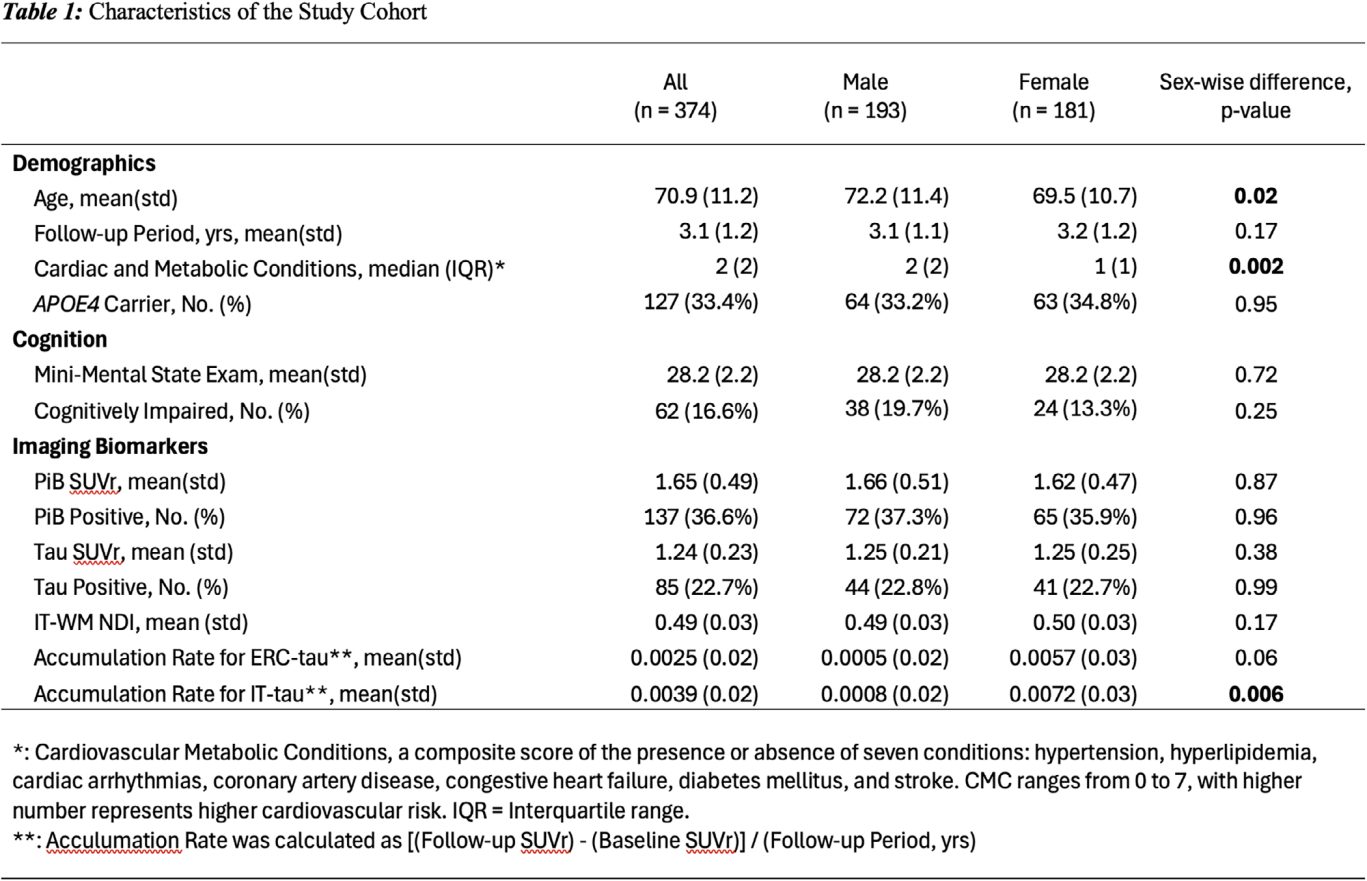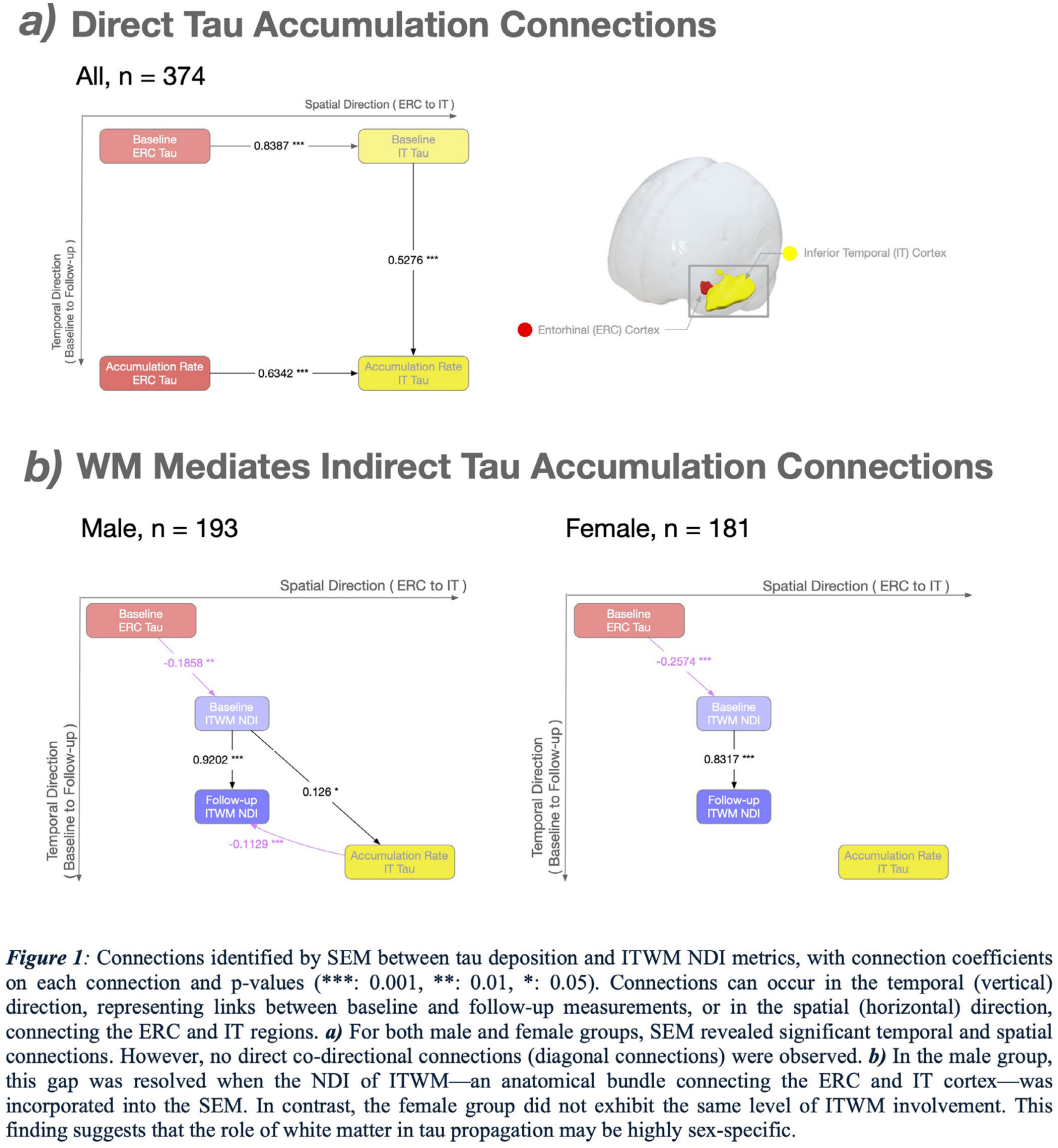# White Matter and Tau Pathology: Evidence for Propagation and Deterioration

**DOI:** 10.1002/alz70856_103781

**Published:** 2025-12-26

**Authors:** Jianqiao Tian, Wen Zhang, Sheelakumari Raghavan, Robert I. Reid, Scott A. Przybelski, Jonathan Graff‐Radford, Val J Lowe, Kejal Kantarci, David S. Knopman, Ronald Petersen, Clifford R. Jack, Prashanthi Vemuri, Christopher G Schwarz

**Affiliations:** ^1^ Mayo Clinic, Rochester, MN, USA; ^2^ Mayo Graduate School of Biomedical Sciences, Rochester, MN, USA; ^3^ Department of Radiology, Nanjing Drum Tower Hospital, Affiliated Hospital of Medical School, Nanjing University, Nanjing, Jiangsu Province, China; ^4^ Department of Quantitative Health Sciences, Mayo Clinic, Rochester, MN, USA; ^5^ Department of Neurology, Mayo Clinic, Rochester, MN, USA; ^6^ Department of Radiology, Mayo Clinic, Rochester, MN, USA

## Abstract

**Background:**

White matter (WM) plays a dual role in interacting with tau pathology. There is an intricate dynamic relationship with WM acting as highways for tau spreading, while the increasing tau burden ultimately compromises WM health. We aimed at studying the associations between tau accumulation and WM integrity using longitudinal data with focus on sex differences.

**Method:**

We included 374 participants who had longitudinal multishell diffusion MRI and flortaucipir PET (baseline mean age = 70.9 years, 52% male, 17% MCI/AD dementia) scans from the Mayo ADRC and Mayo Clinic Study of Aging. Rate of tau accumulation was calculated from the entorhinal cortex (ERC) and inferior temporal cortex (IT) SUVRs, with IT‐WM serving as the WM highway between the two regions. IT‐WM was defined by JHU “Eve” WM atlas, and its integrity was assessed using neurite density index (NDI), which reflects the packing density of neurites. Structural equation modeling (SEM) evaluated relationships between tau and WM metrics.

**Result:**

At baseline, males and females showed no significant differences in tau SUVR or IT‐WM NDI (*p* >0.05), though males had higher vascular risk and averaged two years older than females (Table 1). Figure 1a showed that baseline ERC‐tau influenced baseline IT‐tau, as well as both rate of ERC‐tau and baseline IT‐tau influenced rate of IT‐tau. After incorporating IT‐WM NDI as a mediator (Figure 1b), we discovered 1) baseline ERC‐tau influencing rate of IT‐tau accumulation through baseline IT‐WM NDI, suggesting the role of WM health in the rate of tau accumulation; 2) Rate of IT‐tau accumulation negatively affected follow‐up IT‐WM NDI suggesting the deterioration of WM health as tau accumulation progresses. These relationships were stronger in males compared to females. We also observed greater impact of vascular risk on WM health in males and greater rates of IT‐tau accumulation in females.

**Conclusion:**

Our work sheds light on the mechanistic role of intact WM in tau accumulation (through dense microtubules and synaptic connections) and the deterioration of WM that is associated with higher rates of tau accumulation. We found interesting sex differences that warrant further investigation.